# Understanding 30-Day Mortality After First STEMI Through DAGs: Unravelling Epidemiological Cause-Effect Links

**DOI:** 10.7759/cureus.86178

**Published:** 2025-06-16

**Authors:** Anubha Gupta, Srijan Arora, Manu K Shetty, Amulya Agrawal, Aniket Chauhan, Shekhar Kunal, Girish M Palleda, Lalit Gupta, Dixit Goyal, Mohit D Gupta

**Affiliations:** 1 Center of Excellence in Healthcare, Indraprastha Institute of Information Technology Delhi (IIIT-Delhi), New Delhi, IND; 2 Pharmacology and Therapeutics, Maulana Azad Medical College, New Delhi, IND; 3 Cardiology, Employees' State Insurance Corporation Medical College and Hospital, Faridabad, IND; 4 Cardiology, Govind Ballabh Pant Institute of Postgraduate Medical Education and Research, New Delhi, IND; 5 Anesthesiology, Maulana Azad Medical College, New Delhi, IND

**Keywords:** ai in cvd, ai in epidemiology, directed acyclic graph (dag), in-hospital mortality, notears, stemi

## Abstract

Background and aim: Traditional statistical tests have limitations in analyzing cause-and-effect relationships. Directed acyclic graphs (DAGs) offer a structured representation of causality. This study aimed to utilize DAGs to explore the causal impact of epidemiological factors on 30-day mortality among patients following their first acute ST-elevation myocardial infarction (STEMI).

Method: The study employs data from the North India (NORIN)-STEMI study registry, comprising 3,192 first-time STEMI patients collected prospectively from two tertiary care hospitals in Delhi, India. Continuous optimization structure learning using the Non-combinatorial Optimization via Trace Exponential and Augmented Lagrangian for Structure Learning (NOTEARS) method is applied to learn the DAG. Additionally, a permutation testing framework is proposed for the statistical validation of the links of the DAG.

Results: Among 2,946 first-time STEMI patients, 246 (7.7%) experienced mortality during the study period. A t-test revealed that age was significantly different between the survival and mortality groups within 30 days post-STEMI (p<0.0001). Patients who died within 30 days had a higher mean age (59.90±13.89 years). Furthermore, the study identified a statistically significant association between mortality and HbA1c, triglycerides, smoking, sex, education, occupation, socioeconomic status, physical activity, overall stress, and hypertension.

Conclusion: Our DAG reveals causal relationships and identifies confounding variables affecting mortality after STEMI. Sex is identified as a significant factor influencing mortality both directly and indirectly. This influence occurs through its effects on age, alcohol consumption, stress, hypertension, and socioeconomic status. Additionally, sex is recognized as a confounding factor whose impact on mortality is modified by other factors.

## Introduction

Cardiovascular diseases (CVDs) are a leading cause of early mortality and morbidity worldwide. In 2021, 20.5 million people died from CVDs, with ischemic heart disease and stroke among the top five causes of global disability-adjusted life years (DALYs) in 190 countries and territories [1]. In India, CVDs account for over 30% of deaths, with an age-standardized death rate significantly higher than the global average. The mortality percentage of CVD is decreasing in developed nations because of improved health systems and public health strategies. Low- and middle-income countries account for nearly 80% of this disease burden. Case fatality attributable to CVD in India is much higher than in developed countries. Among all CVD deaths in India, 83% of mortalities are due to ischemic heart disease (IHD) and stroke. In particular, ST-segment-elevation myocardial infarction (STEMI) has a higher in-hospital mortality rate than non-ST-segment-elevation acute coronary syndrome [2].

While conventional risk factors, such as diabetes, hypertension, and dyslipidemia, are well recognized, their interaction with social and behavioral determinants (e.g., stress, education, access to care) complicates risk assessment in STEMI [3]. These complexities are heightened in low- and middle-income countries like India, where healthcare inequities and limited access to timely interventions amplify case fatality [2]. Addressing STEMI necessitates an understanding of the interplay between social, biological, and environmental factors, highlighting the importance of multi-sector interventions to alleviate healthcare system burdens and expenditures and to guide public policies that provide benchmarks for clinical and community-level decisions.

The advent of big data technologies has expanded access to electronic health records (EHRs), enabling large-scale population studies at reduced costs and participant burdens, and reduced selection bias. Despite these advancements, often only the observational data are available, particularly in situations where randomized controlled trials (RCTs) are impractical or unethical [4]. Thus, although an RCT is the best method to analyze the causes of mortality in CVD, in most cases, the mortality and causal effects in STEMI patients have been studied through observational studies [5]. However, in such cases, correlation does not imply causation. Traditional statistical methods, such as multivariable regression models, face limitations in analyzing causal effects [6]. In the light of these challenges, graphical causal models offer a promising approach to studying causal relationships, particularly when observational data are the primary information available. By providing a structured representation of cause and effect, directed acyclic graphs can aid in inferring complex causal pathways, thereby advancing our understanding of mortality determinants in STEMI patients [7].

A directed acyclic graph (DAG) serves as a specialized causal diagram, depicting relationships between features through directed edges or arrows that denote causal effects. By leveraging DAGs, it is feasible to pinpoint factors influencing a given outcome, such as mortality in our context. For instance, research has demonstrated the utility of DAGs in discerning pertinent factors for constructing predictive models in statistical risk analysis [8]. Similarly, in a study analyzing genomics data related to 13 cardiovascular diseases, Yazdani et al. utilized DAGs to unveil risk factors, highlighting their efficacy in inferring causal relationships within complex datasets [9].

Causal networks derived from DAGs are helpful in identifying both direct and indirect risk factors that contribute to disease causation. In CVD, these networks can enable the generation of causal inferences related to epidemiological factors and cardiovascular diseases. For instance, Thornley et al. identified various factors influencing cardiovascular diseases, including sex, age, diabetic status, family history of coronary artery disease (CAD), smoking, drug usage, blood pressure, and cholesterol levels [10]. Additional factors may include dietary habits, socioeconomic status, stress levels, and sleep patterns. Particularly, individuals with obesity are more susceptible to CAD and type 2 diabetes, thereby increasing their risk of developing CVD [11]. Empirical evidence from studies, such as the Framingham prospective cohort study, highlights the correlation between elevated serum cholesterol levels and increased CVD risk [12]. Building a DAG on the CVD features can help in understanding features responsible for early mortality post-STEMI, which can guide clinical decision-making.

However, with the presence of limited and imbalanced data, the DAG connections may need further validation. In this work, we propose using permutation testing for the statistical validation of the connections within these DAGs. Permutation testing is a non-parametric statistical method used to determine the significance of observed data by comparing it to the distribution of data obtained by rearranging the class labels on the observed data points. Unlike traditional parametric tests, permutation tests do not assume a specific distribution for the data. This is crucial in biomedical research, where data may not follow normal distribution due to variability in biological measurements. Also, permutation testing is very helpful in establishing statistical significance when the available sample size is very small and is robust to outliers and heteroscedasticity, which are common issues in biomedical datasets. Due to this, permutation tests find great use in genomics research [13], neuroscience, etc. [14]. Permutation tests have also been shown to be robust at popular significance levels (such as 5%) where traditional tests fail [15]. Surrogate data analysis, the equivalent of permutation tests for time-series data, also finds use in functional brain analysis [14].

Inspired by the above literature, this work utilizes the concepts of directed acyclic graph learning to learn the causal relations between 30-day mortality and epidemiology factors in CVD. The key highlights of this study are as follows: analyzed data from 3,192 STEMI patients across 28 epidemiological variables using t-tests for continuous features and chi-square tests for categorical features for assessing their statistical significance in relation to 30-day mortality. Learned the causal structure using the NOTEARS algorithm. Validated DAG connections through permutation testing - a novel application in this context. Derived clinically relevant inferences and suggested preventive strategies based on validated causal pathways.

## Materials and methods

**Figure 1:**
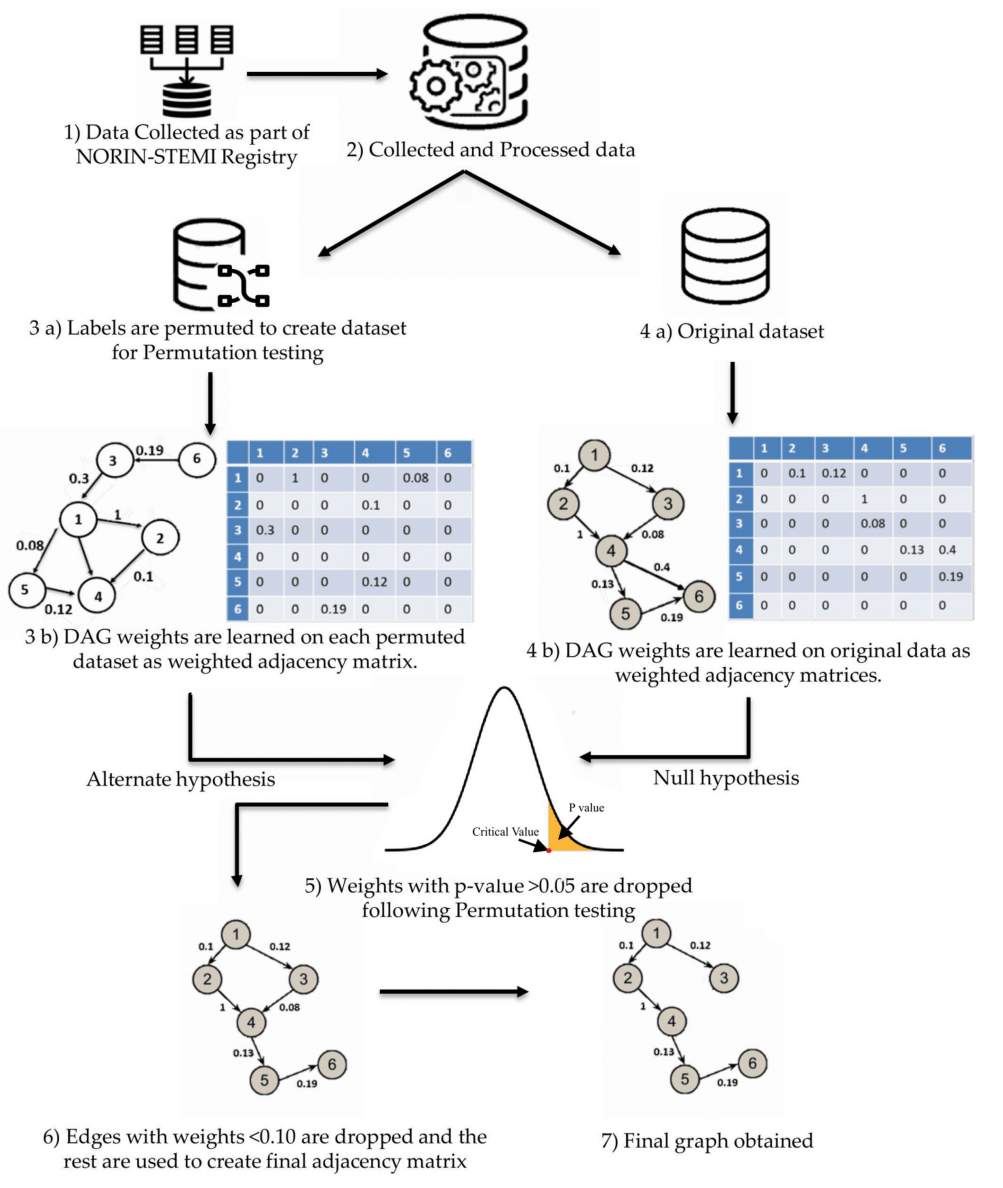
Overview of the workflow for the creation of DAG. NORIN-STEMI: North India ST-segment elevation myocardial infarction registry; DAG: directed acyclic graph. The image is created by the authors of this study.

This study employs data from the NORIN-STEMI study registry, comprising 3,192 first-time STEMI patients collected prospectively from two tertiary care hospitals in Delhi, India [16-18]. The dataset comprises data from 3,192 patients with 28 epidemiology features. The data include both categorical and continuous features. Categorical features included sex, hypertension, hyperlipidemia, uncontrolled diabetes, presentation, education, occupation, diet, cooking oil, socioeconomic status, insurance status, family history of CAD, smoking, physical activity, average sleep duration, overall stress, alcohol, walking, mode of transport, mode of family trip, reason for delay (in reaching the hospital), and location of the first evaluation. The continuous features include age, HbA1c, cholesterol, low-density lipoprotein (LDL), high-density lipoprotein (HDL), and triglycerides. Features were encoded appropriately. Categorical features were either encoded using the ordinal scale or with nominal encoding. Category 0 implies absence, while category 1 implies their presence. After preprocessing, continuous features are discretized using the minimum description length principle algorithm to prepare them for the generation of DAG using NOTEARS [19]. Figure 1 depicts the pictorial representation of the overall methodology.

Data preprocessing

After eliminating redundant features, those with natural ordering ("socioeconomic status," "average sleep duration," "overall stress," "alcohol," "education," and "smoking") were encoded ordinally, ranked according to their real-world values. For example, for "average sleep duration," values of less than 6 hours were encoded as 0, 6-8 hours as 1, and more than 8 hours as 2. This ordinal encoding preserves monotonic relationships of these features, which is essential for causal interpretation in DAG learning. Discrete features without natural ordering were encoded nominally. These features, which lack a natural ranking, were assigned integer values. They include cooking oil, reason for delay, sex, presentation, occupation, diet, insurance status, family history of CAD, physical activity, walking, mode of transport, location of first evaluation, and mode of family trip.

Directed acyclic graph

A directed acyclic graph, as its name suggests, is directed in nature and does not contain any cycles. In other words, by following any path of directed edges on the graph, one cannot return to the starting node. If an edge originates from feature $X_i$ and goes to feature $X_j$, then $X_i$ is called the parent node and $X_j$ is called the child node. DAG serves as a tool for causal studies, with a parent-child relationship denoting a causal relation between them. A node with no outgoing edges denotes a feature that does not affect any other feature, and a node with no incoming edges denotes a feature that is not affected by any other feature. While the problem of learning DAGs remains NP-hard, recent research, such as NOTEARS, formulates the structure learning problem as a continuous optimization problem over real matrices [19]. This leads to the state-of-the-art results without requiring assumptions about the structure of the DAG [19].

NOTEARS algorithm of DAG learning

The NOTEARS algorithm is a method for learning the structure of DAGs in a continuous optimization framework [19]. The goal of NOTEARS is to learn a DAG from data by solving a continuous optimization problem. The key idea is to transform the combinatorial problem of DAG learning for datasets containing a large number of features into a continuous one, which can be solved using proven numerical optimization algorithms having efficient implementations, such as the gradient-based methods. Unlike combinatorial or greedy methods like Peter-Clark (PC) algorithm or Greedy Equivalence Search (GES), NOTEARS imposes a smooth, differentiable acyclicity constraint, enabling the use of gradient-based optimization techniques to learn DAGs efficiently and in a scalable fashion. This makes it particularly suitable for high-dimensional data, where it avoids combinatorial search and allows for end-to-end learning with global optimization, rather than relying on heuristic or local search. Furthermore, the framework is modular and extensible to non-linear and probabilistic models, making NOTEARS a powerful and flexible alternative to conventional constraint-based, score-based, or Independent Component Analysis (ICA)-based methods. As a result, NOTEARS has also been adopted in biomedical studies [20].

Mathematical Formulation

The mathematical formulation of NOTEARS is presented below, outlining its core components and assumptions that enable efficient structure learning through continuous optimization.

1. Input data matrix: Let the input data matrix be $X\in R^{n\times d}$, where d is the number of features and n is the number of samples.

2. Weighted adjacency matrix: Let the weighted adjacency matrix representing the DAG be $W\in R^{d\times d}$, where W_ij_ denotes the weight of the edge from node $j$ to $i$.

3. Linear model: A linear relationship is assumed among the variables, where each variable is modeled as a linear combination of the others plus a noise term. This relationship is represented as below.

 $X = XW + Z$, where $Z$ is the matrix of noise terms.

4. Least squares objective: The objective function to minimize the least squares loss is given by the equation below.

$\min_{W} \frac{1}{2n} ||X - XW||_F^2$, where $||\cdot||$_F_ denotes the Frobenius norm.

5. Acyclicity constraint: The key challenge is to ensure that $W$ represents a DAG. This is achieved by imposing an acyclicity constraint.

$h(W) = \text{trace}\left(e^{W \circ W}\right) - d = 0$, where $\circ$ denotes the Hadamard product (element-wise product) and $e^{W \circ W}$ is the matrix exponential.

6. Optimization problem: Combining the objective and the acyclicity constraint, the resulting optimization problem is formulated as below.

$\min_{W} \frac{1}{2n} ||X - XW||_F^2 \quad$ subject to $\quad h(W) = 0$

To solve this optimization problem, an augmented Lagrangian method is used. The augmented Lagrangian is defined as follows. $\mathcal{L}(W, \alpha, \rho) = \frac{1}{2n}||X - XW||_F^2 + \alpha h(W) + \frac{\rho}{2}h(W)^2$, where $\alpha$ is the Lagrange multiplier and $\rho$ is a penalty parameter.

Algorithm

The algorithm starts by initializing the parameters $W$, $\alpha$, and $\rho$. Then, it iterates until convergence, performing two main steps in each iteration: (a) updating $W$ by minimizing the augmented Lagrangian function $\mathcal{L}(W, \alpha, \rho)$, and (b) updating the Lagrange multiplier $\alpha$ and the penalty parameter $\rho$ to enforce the acyclicity constraint. For the implementation of the NOTEARS algorithm, we used the open-source CausalNex library, available at https://causalnex.readthedocs.io/en/latest/index.html.

Permutation testing

Permutation testing is a statistical technique used to test hypotheses by comparing the observed data with data generated under the null hypothesis. It involves repeatedly shuffling the class labels of the data samples and recalculating the test statistic to create a distribution of the statistic under the assumption of no effect. In our case, the class labels refer to 30-day mortality (survival vs. non-survival). This method allows for assessing the significance of the observed statistic by determining where it falls within this null distribution.

With permutation testing, we aimed to ascertain the statistical significance of causal links of the DAG obtained from the original dataset. Here, we set up a binary hypothesis separately for every link weight recorded in the weighted adjacency matrix. For permutation testing in the context of verifying a link in our learned DAG, the null and alternative hypotheses are framed as described below.

In the context of evaluating the statistical significance of links in the DAG, the null hypothesis ($H_0$) posits that the observed link is not statistically significant and may have arisen by random chance; therefore, it should not be included in the final DAG. In contrast, the alternative hypothesis ($H_1$) asserts that the observed link is statistically significant, indicating a meaningful relationship that warrants its inclusion in the DAG. We carried out permutation testing for every link of the weighted adjacency matrix by following the steps below.

To assess the statistical significance of the edges in the learned graph, a permutation-based approach was employed. The class labels were randomly shuffled while preserving the original label distribution, ensuring that class proportions remained balanced across all permutations. For each permuted dataset, a new weighted adjacency matrix was learned using the NOTEARS algorithm. A total of 100,000 such permuted datasets were generated to represent the null hypothesis. Given the sparsity of the graph and the relatively low incidence of mortality events (~7.7%), a large number of permutations was necessary to ensure the stability of the p-value estimates. After computing p-values for each entry in the original adjacency matrix, all edges with a significance level below 95% (i.e., p >0.05) were considered statistically insignificant and were removed by setting the corresponding entries in the matrix to zero.

Threshold selection

Thresholding allows us to decide the degree of sparsity of the final DAG. The higher the threshold value, the sparser the DAG is. This allows for easier inference, but could also lead to the dropping of important connections. Since the BIC score has been shown to best recover the underlying graph structure among other DAG scoring metrics [21], we plotted the BIC score for different thresholds and found an appropriate value of threshold using the elbow method [22]. The BIC score for graph G on data D with N samples is given by the expression below.

$score_{BIC}(G;D) = LL(G;D) - \frac{log N}{2}dim(G)$, where $LL(G; D)$ denotes the log-likelihood of the DAG when fitted on the data and $dim(G)$ is the model dimension.

Based on this analysis, thresholding was applied with a threshold value of 0.1 to obtain the final non-weighted adjacency matrix (all values <0.1 were set to zero). This threshold was chosen based on the elbow point of the BIC score curve, the inflection where increases in sparsity began to yield diminishing returns in model fit. This allowed for optimal pruning of weak or spurious links while retaining meaningful causal structure and creating the final DAG for inferencing. We used the pgmpy library (https://pgmpy.org/) to fit the DAG to the data and compute the Bayesian Information Criterion (BIC) scores.

## Results

Baseline characteristics

**Figure 2:**
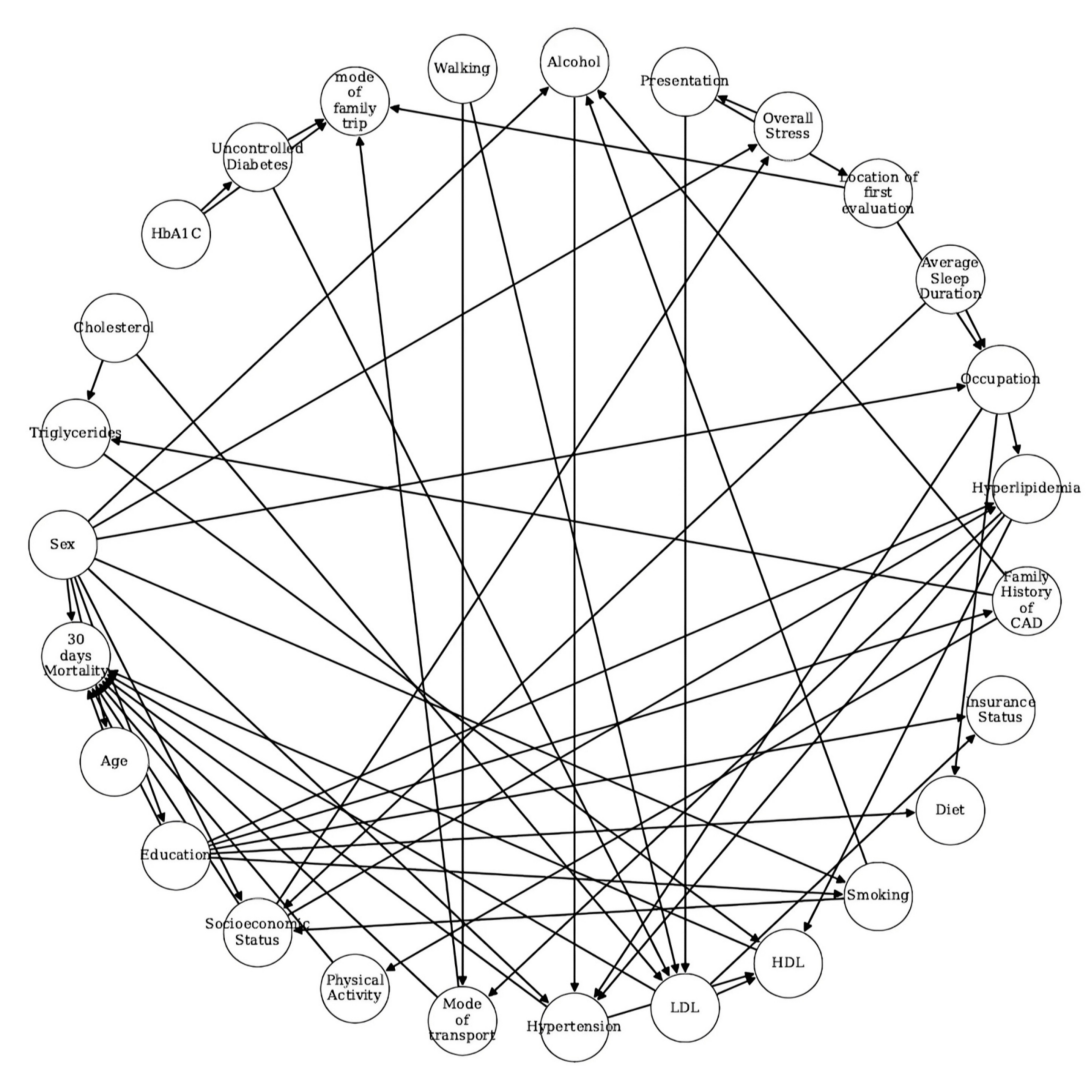
Causal DAG representing different epidemiology factors affecting 30-day mortality, obtained after thresholding and permutation testing. Weights of the direct links from epidemiological variables to 30-day mortality: age (0.553), mode of transport (0.496), sex (0.406), physical activity (0.301), education (0.203), hypertension (0.186), LDL (0.171), HDL (0.166), socioeconomic status (0.145). HbA1c: glycated hemoglobin; CAD: coronary artery disease; HDL: high-density lipoprotein; LDL: low-density lipoprotein; DAG: directed acyclic graph The image is created by the authors of this study.

In this study, we observed that 246 out of 2,946 first-time STEMI patients (7.7%) experienced mortality during the study period. The following sections explain how various factors influenced mortality in these patients. These features include demographic characteristics, personal history, co-morbidities, and other relevant markers. Comparative evaluations are presented for core clinical and background factors (Table 1), lifestyle and behavioral factors (Table 2), and socioeconomic context and healthcare access factors (Table 3). Overall p-values are reported from t-tests for continuous variables (e.g., age) and from global chi-square tests for multi-level categorical variables, comparing distributions between mortality and survival groups. No post-hoc corrections were applied. Using the method described in the previous section, we created DAG as presented in Figure 2.

**Table 1:** Clinical and background features in patients with and without 30-days mortality. HbA1c: glycated hemoglobin; LDL: low-density lipoprotein; HDL: high-density lipoprotein; CAD: coronary artery disease Category 0 implies the absence of hypertension, hyperlipidemia, and uncontrolled diabetes, while category 1 implies their presence.

Features	No mortality (2,946/92.3%)	Mortality (246/7.7%)	p-Value
Age (mean±SD)	53.45±11.21	59.90±13.89	<0.0001
Sex	Female (14.83)	Female (32.93)	<0.0001
	Male (85.17)	Male (67.07)	
HbA1c	6.12±1.60	6.38±1.63	0.00107
Cholesterol	157.82±40.05	157.73±43.12	0.437
LDL	85.34±30.54	88.00±31.85	0.385
HDL	38.26±10.27	36.73±12.14	0.008
Triglycerides	146.96±64.01	138.99±58.76	0.044
Hypertension	0 (72.98)	0 (60.98)	<0.0001
	1 (27.02)	1 (39.02)	
Hyperlipidemia	0 (98.34)	0 (97.15)	0.175
	1 (1.66)	1 (2.85)	
Uncontrolled diabetes	0 (76.82)	0 (67.07)	0.0006
	1 (23.18)	1 (32.93)	
Family history of CAD	No (97.9)	No (95.53)	0.01703
	Yes (2.1)	Yes (4.47)	
Occupation	-	-	Overall (<0.0001)
	Housewife (13.51)	Housewife (29.27)	Housewife (<0.0001)
	Manual laborer (33.44)	Manual laborer (23.17)	Manual laborer (0.00098)
	Professional (11.51)	Professional (6.1)	Professional (0.0094)
	Retired (5.53)	Retired (7.72)	Retired (0.15564)
	Self-employed (26.99)	Self-employed (21.54)	Self-employed (0.06372)
	Student (0.14)	Student (0.81)	Student (0.05703)
	Unemployed (8.89)	Unemployed (11.38)	Unemployed (0.193)
Education	-	-	Overall (0.00639)
	Illiterate (49.15)	Illiterate (58.94)	Illiterate (0.00321)
	Middle school (27.29)	Middle school (26.02)	Middle school (0.67)
	High school completed (16.06)	High school completed (11.38)	High school completed (0.053)
	College graduate (7.5)	College graduate (3.66)	College graduate (0.025)
Socioeconomic status	-	-	Overall (0.01533)
	Lower (25.83)	Lower (22.76)	Lower (0.290)
	Lower middle (32.76)	Lower middle (44.31)	Lower middle (0.0002)
	Upper (0.03)	Upper (0.0)	Upper (-)
	Upper lower (38.73)	Upper lower (30.89)	Upper lower (0.015)
	Upper middle (2.65)	Upper middle (2.03)	Upper middle (0.57)

**Table 2:** Lifestyle and behavioral features in patients with and without 30-days mortality.

Features	No mortality (2,946/92.3%)	Mortality (246/7.7%)	p-Value
Cooking oil	-	-	Overall (0.742)
	Mixed (13.17)	Mixed (11.79)	Mustard (0.509)
	Mustard (82.89)	Mustard (84.55)	Mixed (0.543)
	Sunflower (3.77)	Sunflower (3.25)	Sunflower (0.621)
	Olive (0.17)	Olive (0.41)	Olive (0.686)
Smoking	Non-smoker (36.29)	Non-smoker (43.09)	0.034
	Smoking (63.71)	Smoking (56.91)	
Diet	Non-vegetarian (55.7)	Non-vegetarian (50.41)	0.10848
	Vegetarian (44.3)	Vegetarian (49.59)	
Physical activity	No (86.01)	No (94.72)	0.00011
	Yes (13.99)	Yes (5.28)	
Average sleep duration	-	-	Overall (0.039)
	<6 hours (8.79)	<6 hours (4.06)	<6 hours (0.011)
	6-8 hours (84.52)	6-8 hours (89.03)	6-8 hours (0.058)
	>8 hours (6.73)	>8 hours (6.91)	>8 hours (0.914)
Overall stress	-	-	Overall (0.020)
	Never (22.84)	Never (16.67)	Never (0.026)
	Sometimes (43.69)	Sometimes (42.28)	Sometimes (0.671)
	Several times (33.47)	Several times (41.06)	Several times (0.016)
Alcohol	-	-	Overall (0.246)
	Current - everyday (9.44)	Current - everyday (6.1)	Current - everyday (0.082)
	Current - some days (10.83)	Current - some days (11.38)	Current - some days (0.794)
	Former (4.48)	Former (6.1)	Former (0.248)
	Never (75.25)	Never (76.42)	Never (0.688)
Walking	-	-	Overall (0.224)
	Assisted (0.58)	Assisted (0.81)	Assisted (0.999)
	Unassisted (99.36)	Unassisted (98.78)	Unassisted (0.400)
	Unknown (0.07)	Unknown (0.41)	Unknown (0.247)

**Table 3:** Healthcare access features in patients with and without 30-days mortality. CGHS: Central Government Health Scheme; PCI: percutaneous coronary intervention

Features	No mortality (2,946/92.3%)	Mortality (246/7.7%)	p-Value
Insurance status	-	-	Overall (0.42083)
	CGHS (0.95)	CGHS (0.81)	CGHS (0.87)
	Private insurance (0.68)	Private insurance (0)	Private insurance (-)
	Self-pay (98.37)	Self-pay (99.19)	Self-pay (0.42934)
Reason for delay	-	-	Overall (0.302)
	Lack of transport (9.98)	Lack of transport (13.41)	Lack of transport (0.089)
	Misinterpretation of symptoms (17.14)	Misinterpretation of symptoms (15.04)	Misinterpretation of symptoms (0.402)
	No delay (64.32)	No delay (61.38)	No delay (0.36)
	Patient unwillingness (4.28)	Patient unwillingness (5.69)	Patient unwillingness (0.305)
	Transient resolution of symptoms (4.28)	Transient resolution of symptoms (4.47)	Transient resolution of symptoms (0.893)
Presentation	Direct (5.7)	Direct (5.28)	0.78532
	Referral (94.3)	Referral (94.72)	
Mode of transport	-	-	Overall (<0.00001)
	Ambulance (4.65)	Ambulance (14.63)	Ambulance (<0.0001)
	Public transport (36.39)	Public transport (43.5)	Public transport (0.027)
	Self/family (58.96)	Self/family (41.87)	Self/family (<0.0001)
Location of first evaluation	-	-	Overall (0.0019)
	Non-PCI center	Non-PCI center	Non-PCI center
	Clinic (16.7)	Clinic (16.67)	Clinic (0.994)
	Non-PCI center	Non-PCI center	Non-PCI center
	Hospital (77.26)	Hospital (71.54)	Hospital (0.042)
	PCI center (6.04)	PCI center (11.79)	PCI center (0.0260)
Mode of family trip	-	-	Overall (0.0002)
	Bike (17.99)	Bike (13.01)	Bike (0.049)
	Car (36.05)	Car (26.83)	Car (0.0037)
	On foot (0.85)	On foot (0.0)	On foot (-)
	Scooter (0.07)	Scooter (0.0)	Scooter (-)
	Self/family (43.14)	Self/family (59.35)	Self/family (<0.0001)
	Tractor (0.03)	Tractor (0.0)	Tractor (-)
	Walking (1.87)	Walking (0.81)	Walking (0.293)

Demographic Features

The mean age of individuals who experienced mortality was significantly higher at 59.90±13.89 years compared to the overall study population’s mean age of 53.45 years (mean difference ~6.5 years). This difference was statistically significant (p<0.0001), indicating that older age is associated with a higher risk of mortality following STEMI. The group with mortality was predominantly male (67.07%), with females making up 32.93% (67% vs. 33%). The significant difference in STEMI incidence between genders underscores a higher prevalence of STEMI among males in our cohort.

The educational background of the participants varied widely. A substantial portion of the population was illiterate (58.94%), followed by those with middle school education (26.02%), high school completion (11.38%), and college graduates (3.66%). Illiteracy emerged as a significant factor associated with STEMI, suggesting potential socioeconomic influences on cardiac health. Occupationally, manual laborers constituted a significant portion (23.17%) of the study group, followed by self-employed individuals (21.54%), housewives (29.27%), and professionals (6.1%). These diverse occupational profiles emphasize the broad impact of STEMI across different job categories.

Diet pattern shows minimal variation, with only a slight difference of around 5% in different categories across classes. The study also examined variations across socioeconomic status. It was observed that 25.83% of the survival class belonged to the lower status, 32.76% to the lower middle, 0.03% to the upper status, 38.73% to the upper lower, and 2.65% to the upper middle status. When comparing these with the socioeconomic status of mortality class, it was found that 22.76% were from the lower status, 44.31% from the lower middle, 30.89% from the upper lower, and 2.03% from the upper middle status. The p-value of 0.015 indicates a statistically significant difference in socioeconomic status between survival and mortality classes. Other features, such as cooling oil and insurance status, were not significant between the classes.

Personal History Features

Our study demonstrated the impact of smoking on patient outcomes. The percentages reported represent proportions within the survival and mortality groups. Among smokers, 63.71% were in the survival group, while 36.29% were in the mortality group. Conversely, among non-smokers, 43.09% survived and 56.91% did not. Statistical analysis (p=0.034) confirmed a significant association between smoking status and mortality in STEMI patients. Regarding alcohol consumption, daily drinkers had a survival rate of 9.44% and a mortality rate of 6.1%. Occasional drinkers showed similar proportions as follows: 10.83% survived and 11.38% did not. Former drinkers had 4.48% survival and 6.1% mortality. Most non-drinkers (75.25%) survived, with 76.42% not facing mortality. The lack of statistical significance (p=0.25) may reflect small subgroup sizes and overlapping survival distributions, limiting power to detect true differences.

In sleep pattern analysis, sleeping less than 6 hours or more than 8 hours was associated with higher mortality, while those sleeping 6-8 hours had better survival (84.52%) compared to non-survivors (89.03%). Although this pattern suggests a U-shaped relationship seen in prior cardiovascular studies, these are crude comparisons without adjustment for age or comorbidities.

In the present study, we found a significant link between physical activity and survival rates. Specifically, 86.01% of individuals who were physically active survived, compared to 94.72% of those who were not active and ended up with mortality. On the other hand, 13.99% of participants who engaged in physical activity survived, showing a notable difference in survival percentages (p=0.0001). For overall stress levels, those who survived reported experiencing stress at varying frequencies as follows: 22.84% never, 33.47% several times, and 43.69% sometimes. Similarly, patients who did not survive reported stress percentages of 16.67% never, 41.06% several times, and 42.28% sometimes. These differences were statistically significant (p=0.020). The small absolute differences across stress levels suggest cautious interpretation, as these differences may not translate into clinical relevance.

In sleep pattern analysis, sleeping less than 6 hours or more than 8 hours was associated with higher mortality, while those sleeping 6-8 hours had better survival (84.52%) compared to non-survivors (89.03%). Although this pattern suggests a U-shaped relationship seen in prior cardiovascular studies, these are crude comparisons without adjustment for age or comorbidities.

Comorbidity Features

In the present study, we found significant associations between certain health conditions and survival post-STEMI. Individuals with hypertension had a lower survival percentage (27.02%) compared to those without hypertension (72.98%), thus, having ~2.5x higher prevalence in the mortality group. The difference was highly significant with a p-value less than 0.0001.

For hyperlipidemia, there was a slight difference in survival between individuals with (1.66%) and without (98.34%) the condition. However, this difference was not statistically significant (p=0.17481). Regarding uncontrolled diabetes, individuals with this condition had a lower survival (23.18%) compared to those without uncontrolled diabetes (76.82%). This difference was also significant, with a p-value of 0.0006.

Other Features

In the present study, we examined several blood markers to understand their impact on survival post-STEMI. Average HbA1c was slightly higher in the mortality group (6.38 vs. 6.12; p=0.01835), supporting the link between glycemic control and STEMI outcomes.

Total cholesterol levels were nearly identical between groups (p=0.97). LDL cholesterol was slightly higher in the mortality group (88.00 vs. 85.34; p=0.20793), but this difference was not statistically significant. HDL cholesterol was slightly lower in the mortality group (36.73 vs. 38.26), with a borderline p-value of 0.05437, suggesting a trend that warrants further investigation in larger samples. Interestingly, triglyceride levels were lower in the mortality group (138.99 vs. 146.96; p=0.043). While statistically significant, this counterintuitive finding may reflect metabolic depletion or malnutrition in critically ill patients and should be interpreted with caution.

Other Significant Factors

Additional variables, such as mode of transport, location of first evaluation, and whether the patient traveled with family, were also found to be statistically significant. These reflect access and delay factors, which may indirectly influence STEMI outcomes.

## Discussion

In our investigation into causal relationships and to address the confounding factors between 30-day mortality and epidemiology factors in patients with STEMI, we employed a directed acyclic graph to dissect the intricate relationships among various factors. While statistical significance, particularly with p-values below 0.05, highlights key features, it’s imperative to recognize that statistical significance alone doesn’t reveal direct or indirect impacts on mortality [23]. Here, p-values were used for initial screening, while DAGs provided insight into causal structures, identifying direct, indirect, and confounded relationships. Traditional gold standard research designs, such as randomized controlled trials and observational studies, face challenges in identifying confounding variables [24]. On the other hand, DAG provides causal relationships between variables and also helps in identifying potential confounding variables. DAG identifies factors that can contribute to 30-day mortality after STEMI, either directly or indirectly. The links connecting different features and their directions indicate which features are related to others and their influence or causation. For example, a link between "uncontrolled diabetes" and "LDL" indicates that they are related. It is known that uncontrolled diabetes can lead to dyslipidemia, a condition characterized by abnormal lipid levels, including elevated LDL cholesterol. Poorly controlled blood glucose levels can affect lipid metabolism, leading to increased LDL levels. Thus, a directed link from "uncontrolled diabetes" and "LDL" indicates causation of high LDL by uncontrolled diabetes as observed in our DAG.

In a directed acyclic graph, identifying confounding factors involves examining the pathways between the exposure (independent variable), outcome (30-day mortality), and other variables. Confounding factors are those that are associated with both the exposure and outcome, creating a spurious relationship between them. To identify confounders in a DAG, we need to find the variables that are connected to both the exposure and the outcome but are not intermediaries or mediators, where a mediator lies on the causal pathway between an exposure and the outcome. For example, in the DAG of Figure 2, we find a causal path "sex → alcohol → hypertension → mortality" and another path "sex → mortality," which indicates that sex influences both hypertension (a risk factor) and 30-day mortality, acting as a confounder. Similarly, alcohol and hypertension act as mediators because they fall in the causal path between sex (independent variable) and 30-day mortality. Similar to alcohol consumption, occupation can also indirectly influence mortality through its impact on hypertension. For example, the pathway “sex → occupation → hypertension → mortality” illustrates an indirect effect of occupation on mortality, whereas hypertension affects mortality directly. These examples illustrate how DAGs help differentiate between direct, indirect, confounded, and mediated relationships.

Building upon prior research by Reckelhoff, which established a relationship between sex, hypertension, and mortality, our current DAG reaffirms these findings [25]. Sex directly impacts alcohol consumption, which subsequently influences 30-day mortality indirectly via its association with hypertension. Additionally, sex influences occupation, which in turn affects hypertension. Sex influences socioeconomic status, which in turn influences mortality. Sex also impacts overall stress, which impacts mortality mediated by other variables (presentation and LDL). These interconnected relationships shed light on how these features collectively impact mortality post-STEMI.

Other features, including age, education, socioeconomic status, physical activity, mode of transport, LDL, and HDL, are directly connected to mortality (i.e., outcome variable in the DAG). Among all these features, age emerged as a determinant with a strong and direct impact (weight: approximately 0.553) on mortality. In our DAG for CVD mortality based on the epidemiological data, a link was observed where sex influences the age distribution, and age, in turn, directly impacts mortality. Age is associated with both the exposure (sex) and the outcome (mortality), particularly in the female population, potentially amplifying the risk of mortality as age increases. This highlights the role that higher age plays directly in determining mortality outcomes. The above results are in line with the earlier studies [25-28].

Additionally, our analysis revealed a significant association (link weight of 0.496) between individuals’ mode of transportation and the risk of mortality within 30 days. This underscores the importance of considering transportation methods, as delays in reaching the hospital can lead to treatment delays, potentially impacting mortality outcomes. This is to note that features such as mode of transport and location of first evaluation likely act as proxies for healthcare access and time-to-treatment delays, both of which can influence outcomes in acute STEMI. For instance, delayed referral from a non-specialist or distant center may reflect system-level barriers rather than patient characteristics.

Furthermore, the level of physical activity (link weight of approximately 0.301) was identified as a direct influential factor in 30-day mortality, underscoring the impact of biological and lifestyle factors on mortality within a month’s time frame. Education level is interconnected with smoking, diet, insurance status, family history of cardiovascular disease, and hyperlipidemia. Smoking, in turn, is linked with alcohol consumption, which subsequently connects to hypertension.

Other features also influence mortality. For example, uncontrolled diabetes significantly impacts HbA1c levels, emphasizing the pivotal role of glycemic control. Additionally, diabetes exerts a notable influence on LDL cholesterol, underscoring the importance of managing lipid profiles. It was confirmed from earlier studies that patients with uncontrolled diabetes, who are either on or off statin therapy, have higher LDL [29]. Importantly, elevated LDL cholesterol is linked to increased 30-day mortality rates. LDL and HDL were independently associated with increased relative risk of CVD mortality [30]. Several variables, including walking behaviour, uncontrolled diabetes, and cholesterol levels, exert direct effects on LDL cholesterol, while LDL influences insurance status. Additionally, hypertension, hyperlipidemia, and triglyceride levels influence HDL cholesterol. LDL and HDL cholesterol have a direct impact on 30-day mortality rates. Concurrently, HDL cholesterol, regulated by LDL levels and factors like hypertension and triglycerides, significantly affects 30-day mortality.

In cases where no significant associations were found (e.g., insurance status or diet type), this may reflect limited variation, unequal subgroup sizes, or health system-level uniformity in emergency response that neutralizes their effect. Such findings highlight both the strengths and limitations of observational data in complex healthcare settings.

These findings have practical implications. For clinical decision-making, modifiable risk factors such as physical inactivity, uncontrolled diabetes, or poor access to transport identified as direct or mediated contributors to mortality can be used to prioritize patients for early intervention. For patient risk stratification, understanding how indirect effects (e.g., stress through socioeconomic status) contribute to outcomes allows a more personalized assessment. For future studies, the identified pathways offer targets for prospective trials and public health interventions, such as transport system improvements or stress reduction strategies that address structural and behavioral determinants.

Finally, permutation testing is justified in our case because of our study’s low event rate (~7.7% mortality), the need for robust edge validation, and its appropriateness in non-parametric causal structure learning with sparse graphs. These claims have been justified because of the corroboration of our results with the existing literature as well as with the existing medical knowledge in CVD.

It is crucial to recognize that the DAG offers a simplified depiction of the intricate relationships among factors influencing mortality, and it is possible to find confounding variables. DAG serves as a valuable tool for grasping the fundamental connections among these factors, particularly understanding these connections in the context of STEMI in India. These directional insights help support the identification of modifiable risk factors and clarify where associations may be due to confounding rather than direct causation. Such insights are pivotal for informing public policies and setting benchmarks for clinical and community-level interventions aimed at preventing and managing STEMI effectively.

Limitations

This study has several important limitations that must be considered when interpreting the findings. As this study is based on retrospective observational data, causal claims cannot be definitively established. DAGs are useful for generating hypotheses and visualizing plausible causal pathways, but do not replace the rigor of randomized controlled trials. The data lack time-stamped events, preventing us from establishing a true temporal order between exposures and outcomes, a key component of causality. All associations are unadjusted, and variables such as body mass index, medication adherence, and genetic risk factors were not available. As a result, residual confounding is likely. The NOTEARS algorithm assumes linear relationships and acyclic structure. These assumptions may not fully capture the complexity of biological and social interactions in real-world data. Modifiability of upstream variables like education or transport access was inferred based on existing domain knowledge and not assessed empirically within this dataset. Social and behavioral variables such as stress, occupation, and literacy were included in the DAG model, but often acted as indirect mediators rather than direct causes. Their interpretation should be viewed as hypothesis-generating, especially where the link to mortality was not direct. The dataset is derived from a specific tertiary care government-run setting in North India, which may limit the applicability of findings to other regions or healthcare systems with different population profiles or infrastructure. While permutation testing was used to validate DAG connections, the threshold for edge inclusion (weight >0.1) was chosen based on model fit criteria and may affect the final graph structure. Although we suggest future use of adaptive DAGs for dynamic risk prediction, our dataset did not include the longitudinal or real-time data required to implement such models. Technical challenges such as computational complexity and data availability must be addressed in future studies.

Despite these limitations, the study provides valuable insights into potential causal pathways contributing to STEMI mortality and highlights practical targets for future research and policy interventions.

## Conclusions

This study offers a new understanding of 30-day mortality in STEMI patients by applying causal inference through directed acyclic graphs (DAGs), highlighting how clinical, socioeconomic, and healthcare access factors may interact to influence outcomes. Rather than viewing risk factors like hypertension or uncontrolled diabetes in isolation, the DAG framework suggests these may be downstream effects of broader, inferred modifiable factors such as educational status, occupational stress, and access to timely medical care. The analysis also indicates that socio-behavioral variables, such as occupation, stress, and transport access, may act as indirect mediators, helping explain sex-based disparities in STEMI outcomes. Notably, delayed hospital presentation directly linked to the mode of transport in the DAG emerged as a key and actionable contributor to higher mortality. While variables like stress and literacy were not directly linked to mortality, their positioning in broader causal pathways points to their potential relevance and supports continued exploration in more granular datasets.

These findings emphasize the need to move beyond a narrow focus on hospital-based treatment and adopt integrated strategies that target both medical and structural determinants of health. Moreover, this approach supports a more comprehensive understanding of how social determinants may impact biological and behavioral risks, shaping patient trajectories well before clinical intervention. For policymakers, this study offers data-informed, hypothesis-generating direction. Targeted strategies such as deploying mobile cardiac units in underserved regions and launching public awareness campaigns on early STEMI symptoms are directly aligned with observed DAG pathways and may help reduce avoidable delays in care. Future research should aim to develop adaptive DAG models for dynamic risk prediction and design multi-level interventions that can be validated through prospective studies. However, the success of such models depends on data quality, variable granularity, and contextual relevance. Expanding this methodology to other cardiovascular and chronic diseases could further enhance its clinical and public health impact. Overall, this work bridges the gap between observational epidemiology and practical healthcare improvement, offering a cautiously interpreted, yet actionable framework for precision public health efforts aimed at reducing preventable cardiovascular deaths and promoting equity in outcomes.
